# Acute Exercise Induces FGF21 Expression in Mice and in Healthy Humans

**DOI:** 10.1371/journal.pone.0063517

**Published:** 2013-05-07

**Authors:** Kook Hwan Kim, Seong Hun Kim, Young-Ki Min, Hun-Mo Yang, Jeong-Beom Lee, Myung-Shik Lee

**Affiliations:** 1 Department of Medicine, Samsung Medical Center, Sungkyunkwan University School of Medicine, 50 Irwon-dong Gangnam-gu, Seoul, Korea; 2 Samsung Advanced Institute for Health Sciences and Technology, Sungkyunkwan University School of Medicine, 50 Irwon-dong Gangnam-gu, Seoul, Korea; 3 Department of Physiology, College of Medicine, Soonchunhyang University, 366-1 Ssang yong-dong, Cheonan, Korea; INSERM/UMR 1048, France

## Abstract

Fibroblast growth factor 21 (FGF21) plays an important role in the regulation of energy homeostasis during starvation and has an excellent therapeutic potential for the treatment of type 2 diabetes in rodents and monkeys. Acute exercise affects glucose and lipid metabolism by increasing glucose uptake and lipolysis. However, it is not known whether acute exercise affects FGF21 expression. Here, we showed that serum FGF21 level is increased in mice after a single bout of acute exercise, and that this is accompanied by increased serum levels of free fatty acid, glycerol and ketone body. FGF21 gene expression was induced in the liver but not in skeletal muscle and white adipose tissue of mice after acute exercise, and further, the gene expression levels of hepatic peroxisome proliferator-activated receptor α (PPARα) and activating transcription factor 4 (ATF4) were also increased. In addition, we observed increased FGF21 level in serum of healthy male volunteers performing a treadmill run at 50 or 80% VO_2_max. These results suggest that FGF21 may also be associated with exercise-induced lipolysis in addition to increased catecholamines and reduced insulin.

## Introduction

Fibroblast growth factor 21 (FGF21) is an endocrine hormone that belongs to the FGF family and is mainly expressed in the liver [1]. FGF21 is also produced in other peripheral tissues such as white/brown adipose tissue (WAT/BAT), pancreas and skeletal muscle [2], [3], [4], [5]. During fasting, FGF21 induction via PPARα is required for the activation of free fatty acid (FFA) oxidation, lipolysis and ketogenesis [6], [7], implying that FGF21 plays an important role in adaptive responses to starvation. In addition, FGF21 has a powerful therapeutic potential for the treatment of type 2 diabetes in rodents and monkeys. Pharmacologic studies have shown that FGF21 administration leads to significant improvement in aggravated metabolic phenotypes such as increased fasting glucose, insulin and triacylglycerol (TG) levels in obese (ob/ob or db/db) mice, Zucker diabetic rats and diabetic rhesus monkeys [8], [9], [10], [11].

Exercise is critical for prevention and treatment of metabolic disorders such as obesity, type 2 diabetes, and atherosclerosis [12]. Many reports showed that skeletal muscle produces and releases a variety of cytokines after exercise (referred to myokines), which act as paracrine or endocrine factors, and modulate beneficial effects on metabolic and physiological responses to exercise [13]. These myokines include interleukine-6 (IL-6), IL-15, brain-derived neurotrophic factor (BDNF) and leukemia inhibitory factor (LIF) [14], [15], [16], [17]. Intriguingly, it was recently shown that FGF21 expression is increased in skeletal muscle of muscle-specific Akt1 transgenic mice which exhibit protection from high-fat diet (HFD)-induced obesity and insulin resistance, indicating the beneficial effects of FGF21 as a myokine in metabolic disorders [2]. We also recently showed that increased FGF21 from skeletal muscle with autophagy deficiency contributes to an improvement of obesity and insulin resistance in muscle-specific *Atg7*-deficient mice fed HFD compared to control mice fed HFD [18].

It was recently shown that increased serum FGF21 level is related to daily physical activity in healthy humans [19]. However, it is not known whether acute exercise affects FGF21 expression. Molecular mechanisms by which exercise causes FGF21 induction have not be elucidated. Here, we showed that a single bout of acute exercise increases serum FGF21 levels in mice and healthy men. In addition, we found that FGF21 expression is increased in the liver but not in white adipose tissue and skeletal muscle of mice with acute exercise, and this increase was accompanied by elevated gene expression of hepatic PPARα and ATF4. These results provide an important insight regarding the relationship between FGF21 induction and acute exercise-induced metabolic changes.

## Methods

### 2.1. Animals and Treadmill Exercise

Male C57BL/6 mice were purchased from Orient-Bio Laboratory (Korea). The treadmill test was performed with 12-week-old male mice using the Exer-6M (Columbus Instruments). Mice were warmed up at a speed of 5 m/min for 10 min. Every subsequent 5 min, the speed was increased by 5 m/min. Then, mice ran at a maximum speed of 25 m/min for 30 min or until exhaustion. Exhaustion was defined as the inability to continue regular treadmill running despite repeated electric prodding. All animal were maintained in a facility accredited by the Association for the Assessment and Accreditation of Laboratory Animal Care International (AAALAC #001003). All animal experiments were conducted in accordance with the protocol approved by the Institutional Animal Care and Use Committee of Sungkyunkwan University School of Medicine (permit number: H-B1-012).

### 2.2. Human Subjects

Thirteen healthy male non-athletic volunteers participated in this study which was conducted in accordance with the Helsinki declaration. The physical characteristics of the study subjects are shown in Table 1. All of the subjects read and signed an informed consent regarding the purpose of the study and the procedures, and were asked to refrain from alcohol consumption, smoking, medication or vigorous physical activity during the testing. The study protocol was approved by the University of Soonchunhyang Research Committee.

**Table 1:** Physical characteristics of subjects (n = 13).

Variables	mean ± SEM
Age (yrs)	22.1±0.3
Height (cm)	173.6±1.4
Weight (kg)	68.4±1.3
Body surface area (m^2^)	1.82±0.2
BMI (kg/m^2^)	21.4±0.5
Body fat (%)	20.3±1.3
VO_2_max (ml⋅kg^−^1⋅min^−1^)	41.7±1.3

BMI (body mass index).

### 2. 3. Exercise Conditions and Monitoring for Human Subjects

This study was conducted in a climate chamber between 2–5 p.m., and the environmental conditions were maintained at 24.5±0.3°C, 50±3.0% relative humidity, and 1 m/sec air velocity. Thirteen subjects underwent a treadmill running (Quinton Medtrack SR 60) for 30 min at 50 or 80% VO_2_max as measured using an expired air gas analyzer (Quark Pulmonary Function Testing Lung Volumes Module 2 ergo, COSMED). One week prior to treadmill test, the physical load (VO_2_ max) was determined by performing a prolonged running on a treadmill (gradually increased from 2 to 16 km/h) until the subject became exhausted. Among thirteen subjects who underwent 50% VO_2_max treadmill, eight subjects completed a treadmill running at 80% VO_2_ max 3 days later, while five did not finish the complete course of the test due to exhaustion. Body composition was measured using a multi-frequency bioelectrical impedance model (InBody 720).

### 2.4. Cell Culture

FaO cells, derived from the H4IIE hepatoma cell line, were obtained from the American Type Culture Collection. Cells were maintained in Dulbecco’s modified Eagle’s medium containing 10% fetal bovine serum at 37°C in a humid atmosphere of 5% CO_2_. For treatment of FFA, palmitic acid or oleic acid (Sigma) was treated to FaO cells for 6 h after conjugation with 2% FFA-free bovine serum albumin (BSA) (Sigma).

### 2.5. RNA Isolation and Real-time Reverse Transcription-polymerase Chain Reaction (RT-PCR)

Total RNA from various cells or tissues was prepared using TRIzol (Invitrogen) and purified using a RNA clean up kit (GeneAll) according to the manufacturer’s instruction. cDNA was synthesized from 2 µg of total RNA by RT using MMLV-RT (Moloney Murine Leukaemia Virus) reverse transcriptase (Promega) and oligo(dT) primer at 42°C for 1 h. An aliquot (1/30 vol) of cDNA was then subjected to PCR amplification using the following primers: ATF3, 5′-tgctgctgccaagtgtcgaa-3′ (forward) and 5′-attctgagcccggacgatgc-3′ (reverse); ATF4, 5′-agcaaaacaagacagcagcc-3′ (forward) and 5′-actctcttcttcccccttgc-3′ (reverse); FGF21, 5′-tacacagatgacgaccaaga-3′ (forward) and 5′-ggcttcagactggtacacat-3′ (reverse); PPARα, 5′-ggatgtcacacaatgcaattcg-3′ (forward) and 5′-tcacagaacggcttcctcaggt-3′ (reverse); L32, 5′-cagtcagaccgatatgtgaa-3′ (forward) and 5′-tagaggacacattgtgagca-3′ (reverse). Real-time RT-PCR was performed using SYBR Green I (Takara) with an ABI Prism 7000 (Applied Biosystems). All expression values were normalized to L32 mRNA level.

### 2.6. Plasmid Constructs

pcDNA3-HA-ATF4 was constructed by inserting a human ATF4 fragment into a EcoRI/XhoI-digested pcDNA3-HA vector. pGL3B-FGF21 (−2078/+129) was constructed by inserting a PCR fragment of human FGF21 promoter into a SacI/BglII-digested pGL3B luciferase reporter. pcDNA3/PPARα has been previously described [20]. Nucleotide sequences of all plasmids were confirmed by automatic sequencing.

### 2.7. Luciferase Assay

FaO cells were plated in 24-well culture plates and transfected with a firefly reporter vector (0.2 µg) and Renilla reporter vector (0.01 µg), together with the indicated expression plasmids (0.2 µg) using JetPEI (PolyPlus) reagent according to the manufacturer’s instruction. pcDNA3 empty vector was added to the transfection to keep the total amount of plasmid DNA equivalent per transfection. After 24 h of transfection, cells were lysed in a cell culture lysis buffer (Promega), and luciferase activity was measured. Firefly luciferase activity was normalized to *Renilla* luciferase activity. All assays were performed at least in triplicate.

### 2.8. Measurement of Serum Metabolites

Human blood samples were collected from an antecubital vein in pyrogen-free vacutainers (Vacutainer systems) containing K_3_EDTA before, immediately after and at 1 h after acute exercise. Blood samples were centrifuged at 3,000 rpm for 10 min at 4°C, and the supernatants were harvested and stored at −80°C until analyzed. Glucose level was measured in separated serum using an automated glucose analyzer (ADVIA 1650, Bayer). Serum FFA level was measured using an automated biochemical analyzer (Hitachi 7180). Serum insulin level was determined using an electrochemiluminescence immunoassay (ECLIA) Roche Kit and an E-170 auto-analyzer (Hitachi). β-hydroxybutyrate level was measured by gas chromatography–mass spectrometry using a HP 6890 gas chromatograph equipped with a model 5973 mass selective detector (Hewlett Packard). Human FGF21 level was measured using a Human FGF21 Quantikine ELISA Kit (R&D Systems).

Mouse blood samples were collected using heparinized capillary glass tubes before and immediately after acute exercise. Blood samples were centrifuged at 3,000 rpm for 10 min at 4°C, and the supernatants were harvested and stored at −80°C until analyzed. Serum FGF21 concentration was measured using a Mouse/Rat FGF21 Quantikine ELISA Kit (R&D Systems). Serum FFA level was determined using a SICDIA NEFAZYME Kit (Shinyang Chemical). Serum glycerol level was measured employing a Glycerol Determination Kit (Sigma). Serum β-hydroxybutyrate level was determined using a β-Hydroxybutyrate Assay Kit (BioVision). Blood glucose concentration was measured using an Accu-Check glucometer (Roche).

### 2.9. Statistical Analysis

The values are expressed as mean ± SEM. Statistical analyses were performed using GraphPad Prism Version 5.02 Software. Wilcoxon matched pairs test was used for comparison of metabolites changes in mice before and after exercise. Mann-Whitney test was employed to compare the changes of gene expression in mouse tissues or FaO cell lines treated with FFA. Analysis of metabolites in human subjects was performed using one-way ANOVA with Newman-Keuls post-hoc test for data showing a normal distribution or Kruskal-Wallis test with Dunn’s post-hoc test for data not showing a normal distribution. *P*-values less than 0.05 were considered to represent statistically significant differences.

## Results

### Acute Exercise Increases Serum FGF21 Level

**Figure 1:**
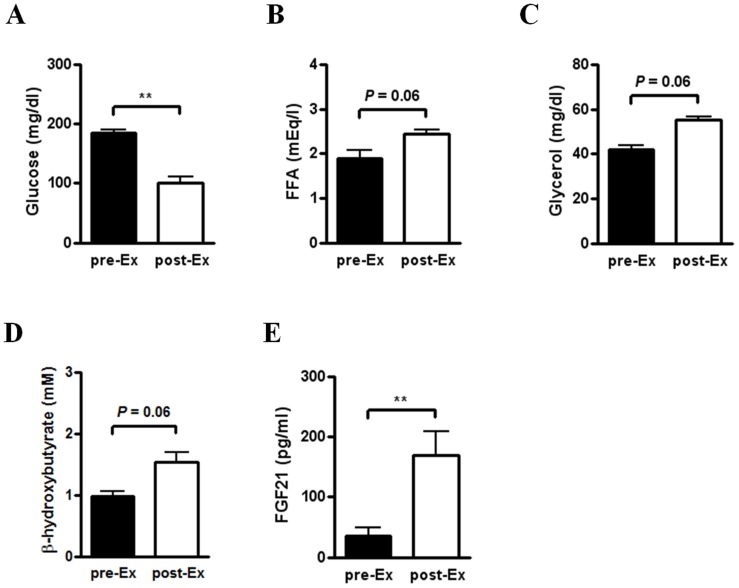
Increased serum FGF21 level in acutely exercised mice. (A–E) Blood or serum parameters in male C57BL/6 mice before (pre-Ex) and immediately after (post-Ex) a single bout of acute exercise with treadmill running. Glucose (n = 8) (A), FFA (n = 5) (B), glycerol (n = 5) (C), β-hydroxybutyrate (n = 5) (D) and FGF21 (n = 8) (E) levels were measured in blood or serum of mice before and immediately after acute exercise for 60 min or until exhaustion. Data are mean ± SEM (**P<0.01 by Wilcoxon matched pairs test).

To study the effect of acute exercise on FGF21 expression, we challenged C57BL/6 mice with a treadmill run for 60 min or until exhaustion. We analyzed plasma parameters of mice before and immediately after a single bout of treadmill exercise. Exercised mice exhibited significantly lower glucose levels compared to mice before exercise (Fig. 1A). Importantly, serum FFA, glycerol and ketone body levels in mice after exercise were higher compared to those before exercise, although the difference was not statistically significant (*P* = 0.06) (Fig. 1B–D). Intriguingly, we observed that serum FGF21 level is significantly increased in exercised mice compared to mice before exercise (Fig. 1E). Therefore, these results suggest that serum FGF21 level is increased in mice showing elevated lipolysis and decreased glucose level after exercise at a high aerobic intensity.

### FGF21 Gene Expression is Increased in the Liver of Acutely Exercised Mice

**Figure 2:**
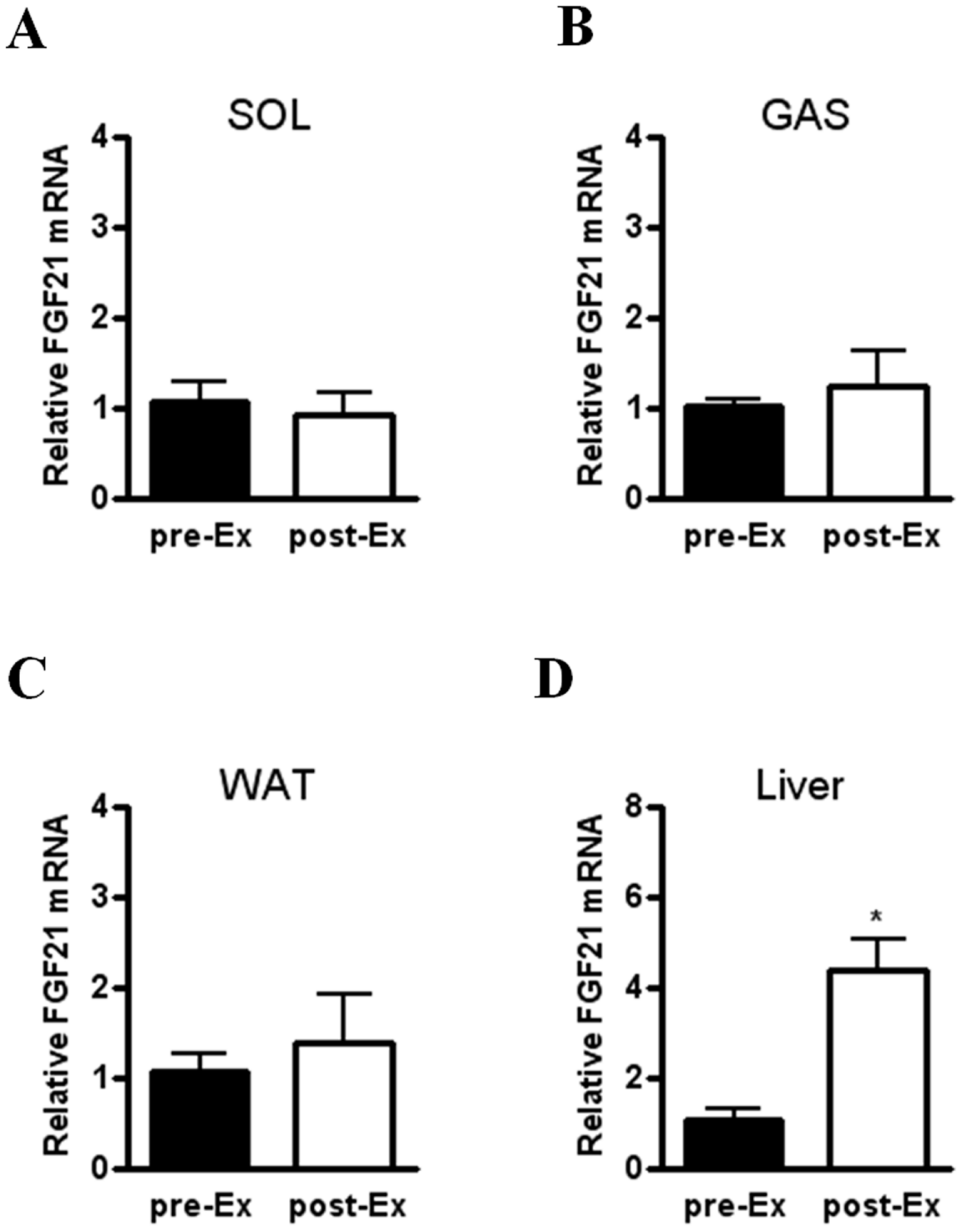
Increased hepatic FGF21 gene expression in acutely exercised mice. (A–D) Relative FGF21 mRNA level in soleus (SOL) (A) and gastrocnemius (GAS) (B) muscle, epididymal WAT (C), and the liver (D) of mice before (pre-Ex) and immediately after (post-Ex) a single bout of acute exercise (n = 4). Data are mean ± SEM (*P<0.05 by Mann-Whitney test).

Because FGF21 has been reported to be expressed in skeletal muscle [2], [5], [18], we next checked FGF21 gene expression in skeletal muscle of acutely exercised mice. Unexpectedly, we found no difference in FGF21 mRNA levels of soleus and gastrocnemius muscle before and after exercise (Fig. 2A and 2B). Because it has been reported that acute exercise affects glucose and lipid metabolism by regulating gene expression or protein activity in the liver and adipose tissue [13], and FGF21 is predominantly expressed in the two organs [1], [4], we tested FGF21 expression levels in the liver and WAT. Hepatic FGF21 mRNA level was significantly elevated in exercised mice compared to mice before exercise, while FGF21 gene expression of WAT was not different between the two groups (Fig. 2C and 2D). Taken together, these findings suggest that increased FGF21 expression in the liver, but not in skeletal muscle and WAT, contributes to elevated serum FGF21 level after acute exercise.

### Acute Exercise-induced ATF4 and PPARα Synergically Increase FGF21 Gene Expression

**Figure 3:**
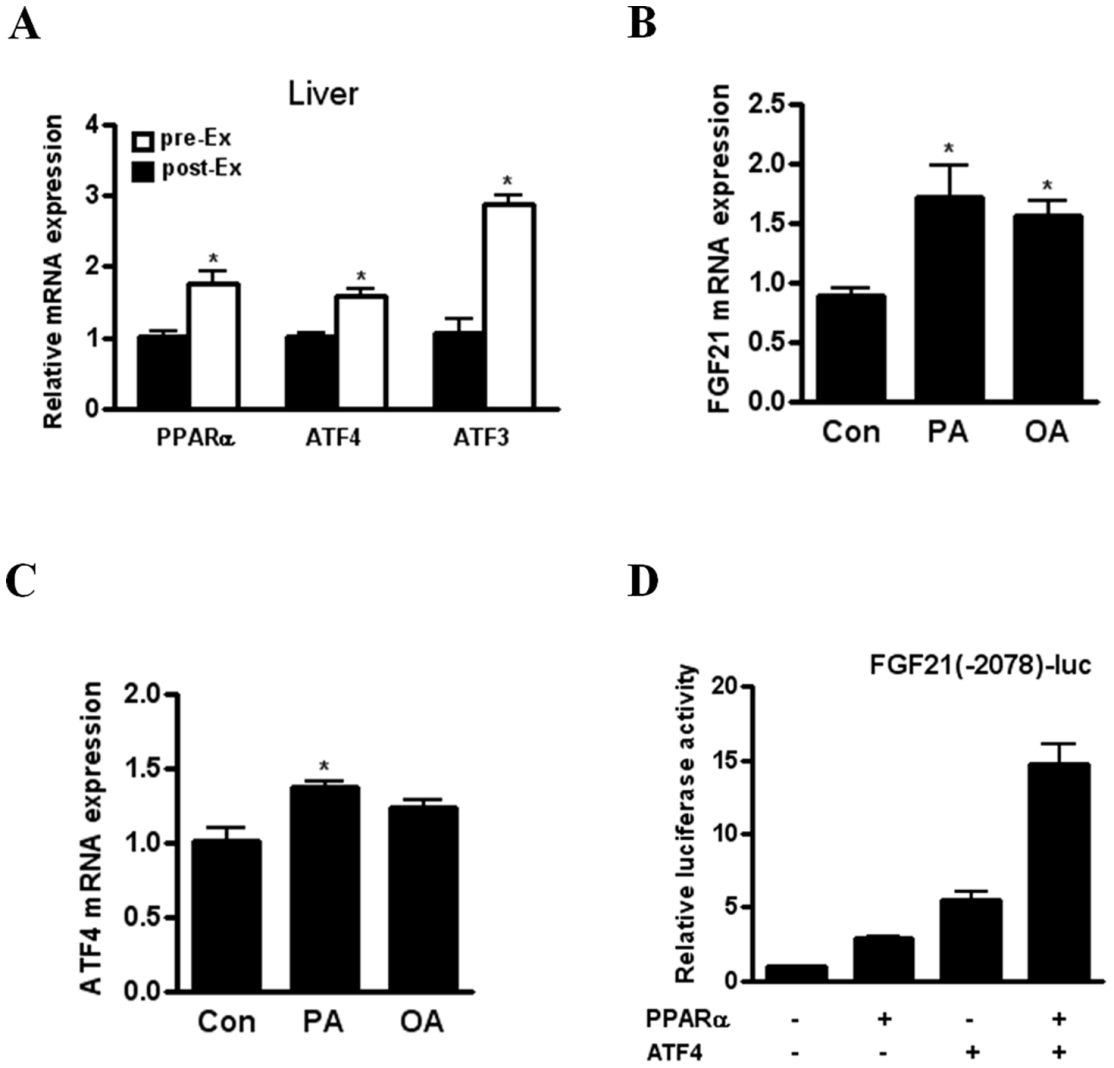
The cooperation of PPARα and ATF4 in increased FGF21 expression after acute exercise or FFA treatment. (A) Relative PPARα, ATF4 and ATF3 mRNA levels in the liver of mice before (pre-Ex) and immediately after (post-Ex) a single bout of acute exercise (n = 4). (B–C) Relative mRNA levels of FGF21 (B) and ATF4 (C) in FaO cells after treatment with 2% FFA-free BSA (Con), BSA-conjugated palmitic acid (400 µM, PA) or BSA-conjugated oleic acid (400 µM,OA) for 6 h (n = 4). (D) Luciferase activity of FGF21 promoter in FaO cells transfected with PPARα or ATF4 for 24 h. Data are mean ± SEM (*P<0.05 and **P<0.01 by Mann-Whitney test).

We next investigated the molecular mechanism by which acute exercise increases the expression of FGF21 gene. ATF3 acts as an adaptive response regulator that is upregulated in response to a variety of stressors such as hypoxia and exercise [21]. We also observed markedly increased ATF3 gene expression in the liver of acutely exercised mice (Fig. 3A). Recently, we showed that ATF4, a key regulator of the integrated response stress, increases FGF21 expression in response to various stresses including autophagy deficiency, mitochondrial stress and amino acid deprivation [18]. Because ATF4 is also known as a transcriptional factor for ATF3 induction [21], we investigated the expression of ATF4 gene in the liver of mice showing increased hepatic FGF21 expression after acute exercise. Importantly, ATF4 gene expression was enhanced in the liver of exercised mice together with elevated gene expression of PPARα, a known positive regulator for FGF21 induction (Fig. 3A). Because FFA released from adipose tissues by exercise-induced lipolysis is transported to the liver and is able to induce FGF21 expression, we next studied the effect of FFA on FGF21 gene expression in FaO cells. Importantly, palmitic or oleic acid increased FGF21 gene expression in FaO cells (Fig. 3B). In addition, we showed that palmitic acid but not oleic acid induces the expression of ATF4 gene in FaO cells (Fig. 3C). Moreover, we found that ATF4 overexpression synergically enhances PPARα-induced luciferase activity of FGF21 promoter (Fig. 3D). Thus, our data suggest that ATF4 and PPARα cooperatively induce FGF21 gene expression in the liver of acutely exercised mice.

### Serum FGF21 Level is Increased in Healthy Subjects after Acute Exercise

**Figure 4:**
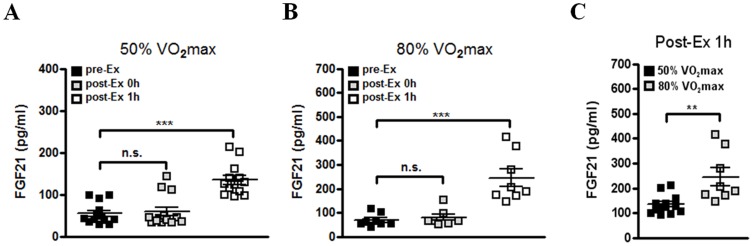
Increased serum FGF21 level in healthy humans after acute exercise. (A–B) Serum FGF21 level in healthy subjects with treadmill running at 50% (n = 13) (A) or 80% VO_2_max (B) (n = 8) for 30 min. FGF21 level was determined in serum of subjects before (pre-Ex), immediately after (post-Ex 0h) or at 1 h after (post-Ex 1h) acute exercise. (C) Serum FGF21 level after 1 h recovery from acute exercise in healthy subjects after a 50% or 80% VO_2_max treadmill running for 30 min (n = 8–13). Data are mean ± SEM (**P<0.01 and ***P<0.001 by Kruskal-Wallis test with Dunn’s post-hoc test (A–B) or Mann-Whitney test (C)). (n.s., not significant).

**Figure 5:**
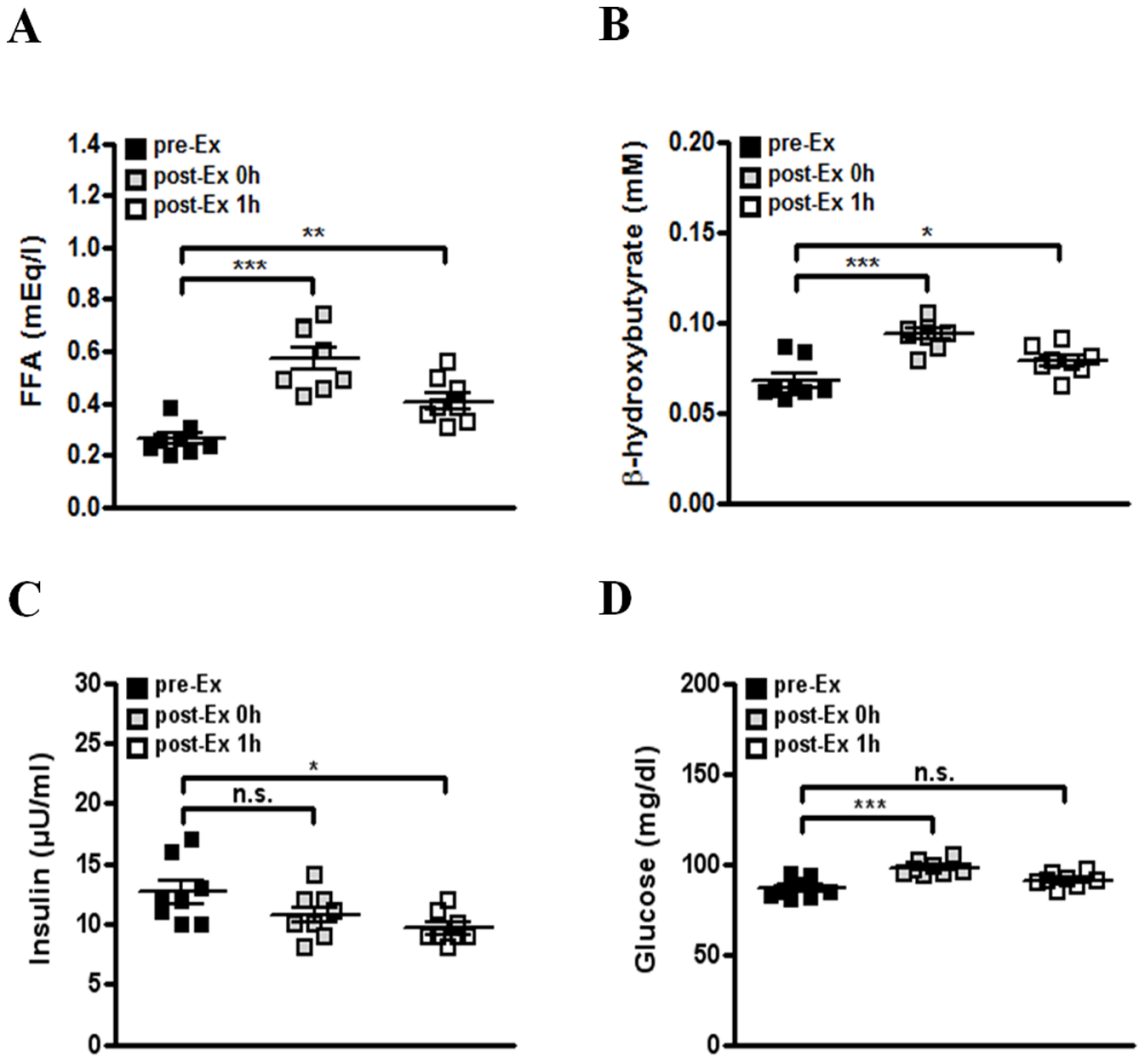
The changes of serum metabolites in human subjects after acute exercise. (A-D) Serum metabolite levels in healthy subjects with treadmill running at 80% VO_2_max (n = 8) for 30 min. Serum FFA (A), β-hydroxybutyrate (B), insulin (C) and glucose (D) levels were determined before (pre-Ex), immediately after (post-Ex 0 h) or at 1 h after (post-Ex 1 h) acute exercise. Data are mean ± SEM (*P<0.05, **P<0.01, ***P<0.001 by one-way ANOVA with Newman-Keuls post-hoc test). (n.s., not significant).

We finally investigated whether acute exercise increases serum FGF21 level in human subjects. A treadmill running test was performed in healthy men according to the Bruce protocol [22]. After the 1 h recovery from acute exercise at 50% or 80% VO_2_max for 30 min, serum FGF21 level was significantly higher than that before acute exercise, while there was no difference in serum FGF21 level between before and immediately after acute exercise (Fig. 4A and 4B). In addition, after the 1 h recovery from high-intensity exercise (80% VO_2_ max), serum FGF21 level was higher than that after the 1 h recovery from mild intensity exercise (50% VO_2_ max) (Fig. 4C), suggesting that FGF21 level may be proportionally increased according to the intensity of acute exercise. Similar to changes of metabolites in exercised mice, serum FFA and ketone body levels were significantly higher in exercised subjects compared to those in rested subjects (Fig. 5A and 5B), implying the increase of exercise-induced lipolysis in human subjects. We also found that serum insulin level after an l h recovery from exercise is lower compared to that before exercise (Fig. 5C). However, serum glucose level of human subjects was not decreased after acute exercise (Fig. 5D). Taken together, our data suggest that a single bout of acute exercise increases serum FGF21 level in healthy subjects together with enhanced lipolysis.

## Discussion

In response to starvation, FFAs released from the adipose tissue by lipolysis are employed as an energy source through fatty acid oxidation in many peripheral tissues or as substrate for ketogenesis in the liver. FGF21 has been reported to play an important role in starvation-induced lipolysis, fatty acid oxidation and ketogenesis [6], [7]. Similar to starvation, acute exercise also promotes lipolysis of adipose tissue, and subsequently released FFAs are utilized as a major fuel for ATP production in peripheral tissues such as skeletal muscle and the liver. Here, we observed that a single bout of acute exercise increases serum FGF21 levels in mice and healthy men. In addition, we demonstrated a correlation between increased FGF21 level and enhanced free fatty acid, glycerol or ketone level after a single bout of acute exercise. We also found that the cooperation of PPARα and ATF4 synergically induces FGF21 gene expression in the liver of acutely exercised mice. Our results suggest that hepatic FGF21 induction after acute exercise may contribute to increased serum FGF21 level.

It was recently reported that serum FGF21 level is increased after 2 weeks of exercise [23]. However, the relationship between acute exercise and FGF21 level is still unknown. In the present study, we first found that acute exercise induces an elevation of serum FGF21 level in mice and humans. Based on the role of FGF21 on glucose clearance, lipolysis and ketogenesis, we speculate that increased FGF21 mediates some of the beneficial effects of acute exercise on glucose and lipid metabolism. Further study is necessary to evaluate the physiological role of FGF21 on acute exercise-induced changes in glucose and lipid metabolism.

In the present study, we showed that acute exercise induces FGF21 expression in the liver but not skeletal muscle and adipose tissues. In addition, we observed increased ATF4 and PPARα gene expression in the liver of acutely exercised mice. We also found that palimtic acid induces ATF4 and FGF21 gene expression in FaO cells, while oleic acid increased gene expression of FGF21 but not ATF4. Based on previous reports showing the positive effect of palmitic acid on ATF4 expression and that of oleic acid on PPARα transcriptional activity [24], [25], we speculate that FFAs released from adipose tissue by acute exercise-induced lipolysis both induce gene expression of ATF4 and enhance the transcriptional activity of PPARα in the liver, leading to the synergistically increased hepatic FGF21 expression.

It has been reported that exercise mediates metabolic benefits via several mechanisms [13]. Among them, it is well known that AMPK plays a crucial role in alterations of energy metabolism during exercise [13]. In the present study, we showed that increased PPARα after acute exercise is involved in elevated FGF21 gene expression, consistent with previous papers showing the role of PPARα as a positive regulator on FGF21 induction [6], [7], [26]. In addition, we found that increased ATF4 activation is associated with acute exercise-induced hepatic FGF21 expression. Based on the present findings and previous papers showing that FGF21 is induced by ATF4 in response to various stresses such as autophagic deficiency, mitochondrial stress and leucine deprivation [18], [27], we hypothesize that ATF4-FGF21 axis plays an important role in the adaptive response to diverse stresses. Further study is needed to evaluate the significance of ATF4-FGF21 axis in other physiological and pathological conditions or models.

FGF21 is a crucial mediator in the adaptive metabolic response to starvation, and is an excellent therapeutic molecule for treatment of type 2 diabetes and obesity in rodents and monkeys. In the present study, we showed that acute exercise leads to the systemic increase of serum FGF21 level in mice and healthy humans, likely due to increased hepatic FGF21 expression. Therefore, our results provide new insights regarding the role of FGF21 in addition to increased catecholamines and reduced insulin in acute exercise-induced lipolysis and metabolic changes. Our findings also suggest a therapeutic strategy to develop exercise-mimetic drugs for treatment of diabetes and obesity.
